# High rates of unfavourable TB treatment outcomes observed in Madang Province, Papua New Guinea

**DOI:** 10.5588/pha.24.0015

**Published:** 2024-09-01

**Authors:** W. Toua, V. Lape, J.W. Bolnga, M. Daimen, T. Kelebi, S. Vaccher, J. Greig

**Affiliations:** ^1^ Madang Provincial Health Authority, Madang, Papua New Guinea;; ^2^ West Sepik Provincial Health Authority, Papua New Guinea;; ^3^ Burnet Institute, Melbourne, VIC, Australia.

**Keywords:** loss to follow-up, tuberculosis, PNG

## Abstract

**SETTING:**

Madang Province is located on the northern coast of Papua New Guinea (PNG), a critical mixing point between the populous highlands and more remote regions. Madang Province faces challenges with limited capacity to diagnose and treat TB.

**OBJECTIVE:**

To describe the TB caseload and investigate factors associated with known unfavourable treatment outcomes.

**DESIGN:**

This is a retrospective cohort study using routinely collected TB programmatic data for treatments commenced 1 January 2019 to 31 December 2021. Using multivariable logistic regression, factors associated with known unfavourable treatment outcomes—death, failure after treatment, and loss to follow-up (LTFU)—were evaluated.

**RESULTS:**

Of the 4,668 registered and treated, 3,755 had an evaluated outcome, and 33% had unfavourable outcomes, most commonly LTFU (23%). Unfavourable treatment outcomes were significantly associated with HIV-untested (aOR 2.82 compared to HIV-negative; 95% CI 2.39–3.33), having drug-resistant TB (aOR 3.26 compared to drug-susceptible TB, 95% CI 1.18–9.00), and travel time to the health facility 1–<3 hours by foot (aOR 3.53 compared to <1 hour by foot; 95% CI 1.04–12.06).

**CONCLUSION:**

High LTFU from TB treatment was associated with factors that indicate barriers to access to care and treatment completion. Decentralisation and strengthening of TB services for improved person-centred care and treatment support are urgently required in Madang Province.

## Abstract (fr)

**CADRE:**

La province de Madang est située sur la côte nord de la Papouasie-Nouvelle-Guinée (PNG), un point de mélange essentiel entre les hauts plateaux peuplés et les régions plus éloignées. La province de Madang est confrontée à des défis avec des capacités limitées pour diagnostiquer et traiter la TB.

**OBJECTIF:**

Décrire le nombre de cas de TB et enquêter sur les facteurs associés aux résultats défavorables connus du traitement.

**METHODE:**

Il s'agit d'une étude de cohorte rétrospective utilisant des données programmatiques de lutte contre la TB collectées en routine pour les traitements commencés du 1^er^ janvier 2019 au 31 décembre 2021. À l'aide d'une régression logistique multivariée, les facteurs associés aux résultats défavorables connus du traitement (décès, échec après le traitement et perte de suivi [LTFU, pour l’anglais « loss to follow-up ») ont été évalués.

**RÉSULTATS:**

Sur les 4668 personnes enregistrées et traitées, 3755 ont eu un résultat évalué et 33% ont eu des résultats défavorables, le plus souvent des LTFU (23%). Les résultats défavorables du traitement étaient significativement associés au fait que les personnes n'ayant pas fait l'objet d'un test de dépistage du VIH (OR ajusté [ORa] 2,82 par rapport aux personnes séronégatives ; IC à 95% 2,39–3,33), ayant une TB pharmacorésistante (ORa 3,26 par rapport à la TB sensible aux médicaments, IC à 95% 1,18–9,00) et le temps de déplacement jusqu'à l'établissement de santé étant de 1 à <3 h à pied (ORa 3,53 contre <1 h à pied ; IC à 95% 1,04–12,06).

**CONCLUSION:**

Un taux élevé dee LTFU provenant du traitement de la TB était associé à des facteurs indiquant des obstacles à l'accès aux soins et à l'achèvement du traitement. La décentralisation et le renforcement des services de lutte contre la TB pour améliorer les soins centrés sur la personne et le soutien au traitement sont nécessaires de toute urgence dans la province de Madang.

TB infection is a leading cause of death globally and remains a major public health problem. In 2022, 7.5 million new TB infections were reported globally, and approximately 18% of cases occurred in the Western Pacific Region, including Papua New Guinea (PNG). PNG is included in the WHO list as a high-burden country for TB and for multidrug-resistant/rifampicin-resistant TB (MDR/RR-TB), with an estimated incidence rate of 432 per 100,000 population.^1^ TB notifications had increased across all regions of PNG before the COVID-19 pandemic, but rates of bacteriological confirmation remained stubbornly low.^2,3^

A challenge facing low-middle-income countries (LMIC) is often related to health system weaknesses in supporting the TB programme, from gaps in governance, workforce, and programme resourcing to supply chain and data management.^4,5^ These challenges, combined with patient socio-demographic and clinical factors, have been related to unsatisfactory treatment outcomes in PNG and other TB-endemic settings.^6–8^ TB caseload and treatment outcomes are major national TB programme (NTP) indicators that influence the implementation of policies and practices.

An ongoing challenge facing PNG is high LTFU and unevaluated TB patient outcomes. These challenges have been previously described in rural parts of Madang Province and Sandaun Province for drug-susceptible (DS) TB.^6,9^ In this study, we aimed to describe caseload and treatment outcomes for DS-TB and MDR/RR-TB treatment in Madang Province and to evaluate factors associated with known unfavourable outcomes.

## METHODS

### Study setting

PNG is situated in the easternmost part of the island of Papua, bordering Indonesia, Australia, and the South Pacific Islands. PNG hosts an estimated population of over 11 million people,^10^ primarily residing in rural and remote areas with substantial linguistic and cultural diversity.^11,12^ Madang Province is situated on the north coast with an estimated total population of 797,807.^10^ The case notification rate for TB in Madang Province was reported as 243 and 333 per 100,000 population in 2016 and 2019, respectively.^3,13^ There are 11 basic management unit (BMU) health facility sites in the province, but only three provide GeneXpert^®^ MTB/RIF (Cepheid, Sunnyvale, CA, USA) assay for diagnosis: Madang Provincial Hospital TB Clinic (also known as Modilon TB Clinic), Gaubin Rural Hospital, and Malala Health Centre.

### Study design and population

A retrospective cohort study was conducted using NTP data routinely collected into treatment registers in 2019–2021 at the three BMU sites that provide Xpert MTB/RIF testing.

### Data collection

The variables collected from TB treatment registers were age, sex, enrolment BMU, TB registration category, HIV status, TB site, TB type, TB confirmation method, treatment regimen, date treatment commenced, date treatment completed, and treatment outcomes. Based on patient home location (residential address) details in registers, facility staff estimated the time required to travel to the BMU and the mode of transport most used from that location. All TB patients who were registered and commenced TB treatment (first- and second-line TB treatment) from 1 January 2019 to 31 December 2021 were included. Diagnosis methods included sputum smear microscopy, Xpert MTB/RIF (detecting both DS-TB and RR-TB) for bacterial confirmation, and clinical diagnosis of pulmonary or extrapulmonary TB. Treatment outcomes for both DS-TB and MDR/RR-TB were assigned based on definitions in the WHO guidelines and PNG National TB Programme guidelines.^14,15^ Outcome cohort reporting timeframes were used for DS and DR treatments. “Unfavourable treatment outcome” was a composite definition that included the reported treatment outcomes of treatment failure, death, and LTFU. Not evaluated was excluded from the main analysis but considered an unfavourable outcome for sensitivity analyses.

### Data analysis

Categorical variables were summarised as frequencies and percentages, and continuous variables as median and interquartile range (IQR). Age was categorised. Categorical variables were compared between groups using the χ^2^ test, and continuous variables were compared using the Kruskal-Wallis test. *P* < 0.05 was considered statistically significant. Risk factors for unfavourable outcomes were estimated as crude odds ratios (ORs) using univariable analysis, with adjusted odds ratios (aOR) calculated through multivariable logistic regression using a forward stepwise approach. Where relevant, interaction terms were assessed and included. Data were analysed using STATA v17 (Stata Corp, College Station, TX, USA).

### Ethics approval

Ethical approval was provided by the PNG Medical Research Advisory Council, Port Moresby, PNG.

## RESULTS

Among 4,668 patients registered for treatment, 50.4% were male (Table 1). The median age was 27 years (IQR 18–40), and 19.1% were children (<15 years of age). Most cases (68.9%) were seen at Modilon TB Clinic, with Malala Health Centre treating only 2.5% (116) of cases across the three BMUs. BMU staff estimated that most people (80.3%) could travel to their BMU within 1 hour, and transportation was most commonly by car (55.1%). Of all treatment registrations, 11.0% were retreatment cases, and 63.1% were pulmonary TB (Table 1). Overall, 62.9% of TB cases were bacteriologically confirmed, but this was only 20.7% (356/1,724) of extrapulmonary TB (EPTB) diagnoses compared to 87.6% (2,578/2,944) of pulmonary TB diagnoses. There were 23 DR-TB cases (0.5% of all registrations; 3.9% (20/515) of retreatment cases). Among the 3,399 cases tested for HIV, only 112 (2.4%) had a positive result; 27.0% of the whole cohort did not have HIV status tested.

**TABLE 1.** Socio-demographic, clinical and programmatic characteristics of TB notifications in Madang Province, PNG, 2019–2021, by BMU facility.

Variables	Total	Modilon	Gaubin	Malala	*P*-value
	(*n* = 4,668)	(*n* = 3,218, 68.9%)	(*n* = 1,334, 28.6%)	(*n* = 116, 2.5%)	
	*n* (%)	*n* (%)	*n* (%)	*n* (%)	
Sex					
Male	2,353 (50.4)	1,638 (50.9)	654 (49.0)	61 (52.6)	<0.001
Female	2,314 (49.6)	1,580 (49.1)	680 (51.0)	54 (46.6)	
Missing	1 (0.0)	0 (0.0)	0 (0.0)	1 (0.9)	
Age, years, median [IQR]	27 [18–40]	26 [18–39]	32 [20–50]	28 [19–43]	<0.001
Age categories, years					
0–4	381 (8.2)	297 (9.2)	79 (5.9)	5 (4.3)	<0.001
5–14	511 (10.9)	387 (12.0)	114 (8.5)	10 (8.6)	
15–34	2,099 (45.0)	1,530 (47.5)	516 (38.7)	53 (45.7)	
35–64	1,538 (32.9)	952 (29.6)	539 (40.4)	47 (40.5)	
≥65	139 (3.0)	52 (1.6)	86 (6.4)	1 (0.9)	
Time to travel to BMU, hours					
<1	3,747 (80.3)	2,610 (81.1)	1,034 (77.5)	103 (88.8)	<0.001
1–<3	793 (17.0)	480 (14.9)	300 (22.5)	13 (11.2)	
≥3	128 (2.7)	128 (4.0)	0 (0.0)	0 (0.0)	
Mode of travel to BMU					
By foot	1,851 (39.7)	1,048 (32.6)	709 (53.1)	94 (81.0)	<0.001
By car	2,572 (55.1)	1,959 (60.9)	591 (44.3)	22 (19.0)	
By boat	23 (0.5)	23 (0.7)	0 (0.0)	0 (0.0)	
Mixed transport	222 (4.8)	188 (5.8)	34 (2.5)	0 (0.0)	
Registration category					
New	4,150 (88.9)	2,865 (89.0)	1,171 (87.8)	114 (98.3)	0.009
Retreatment	515 (11.0)	350 (10.9)	163 (12.2)	2 (1.7)	
Unknown	3 (0.1)	3 (0.1)	0 (0.0)	0 (0.0)	
Site of TB disease					
Pulmonary	2,944 (63.1)	2,003 (62.2)	862 (64.6)	79 (68.1)	0.17
Extrapulmonary	1,724 (36.9)	1,215 (37.8)	472 (35.4)	37 (31.9)	
Type of TB by drug resistance					
DS-TB	3,857 (82.6)	2,407 (74.8)	1,334 (100.0)	116 (100.0)	<0.001
DR-TB	23 (0.5)	23 (0.7)	0 (0.0)	0 (0.0)	
Clinical TB	788 (16.9)	788 (24.5)	0 (0.0)	0 (0.0)	
HIV status					
Positive	112 (2.4)	90 (2.8)	22 (1.6)	0 (0.0)	<0.001
Negative	3,294 (70.6)	2,220 (69.0)	1,012 (75.9)	62 (53.4)	
Not tested	1,262 (27.0)	908 (28.2)	300 (22.5)	54 (46.6)	
TB confirmation method					
Bacteriologically confirmed	2,934 (62.9)	1,774 (55.1)	1,044 (78.3)	116 (100.0)	<0.001
Clinical diagnosis	1,734 (37.1)	1,444 (44.9)	290 (21.7)	0 (0.0)	
TB treatment regimen					
First line	4,645 (99.5)	3,195 (99.3)	1,334 (100.0)	116 (100.0)	0.005
Second line	23 (0.5)	23 (0.7)	0 (0.0)	0 (0.0)	
Year treatment started					
2019	1,506 (32.3)	1,094 (34.0)	376 (28.2)	36 (31.0)	0.003
2020	1,608 (34.4)	1,094 (34.0)	475 (35.6)	39 (33.6)	
2021	1,554 (33.3)	1,030 (32.0)	483 (36.2)	41 (35.3)	

PNG = Papua New Guinea; BMU = basic management unit, IQR = interquartile range; DS-TB= drug-susceptible TB; DR-TB = drug-resistant TB.

Among 3,755 treatments with an evaluated outcome, 2,498 (66.5%) were favourable (cured or treatment completed), with the proportion significantly lower in 2021 (59.4%; *P* < 0.001) (Table 2). For unfavourable treatment outcomes among cases with an evaluated outcome, 47 (1.3%) died, 120 (3.2%) failed after treatment, and 1,090 (23%) were LTFU. Nearly one-fifth (19.6%; 913/4668) of all those who commenced treatment had outcomes assigned as not evaluated.

**TABLE 2.** Characteristics of patients with favourable and known unfavourable TB treatment outcomes in Madang Province, PNG, 2019–2021.

Variables	Total	Favourable	Unfavourable	*P*-value
	(*n* = 3,755) *n* (%)	(*n* = 2,498, 66.5%) *n* (%)	(*n* = 1,257, 33.5%) *n* (%)	
Sex				
Male	1,861 (49.6)	1,211 (48.5)	650 (51.8)	0.058
Female	1,893 (50.4)	1,287 (51.5)	606 (48.2)	
Age, years, median [IQR]	28 [19–41]	28 [19–42]	26 [18–40]	0.007
Age categories, years				
0–4	305 (8.1)	191 (7.6)	114 (9.1)	0.16
5–14	392 (10.4)	258 (10.3)	134 (10.7)	
15–34	1,680 (44.7)	1,101 (44.1)	579 (46.1)	
35–64	1,264 (33.7)	873 (34.9)	391 (31.1)	
≥65	114 (3.0)	75 (3.0)	39 (3.1)	
Enrolling BMU				
Modilon	2,587 (68.9)	1,575 (63.1)	1,012 (80.5)	<0.001
Gaubin	1,133 (30.2)	896 (35.9)	237 (18.9)	
Malala	35 (0.9)	27 (1.1)	8 (0.6)	
Mode of travel to BMU				
By foot	1,505 (40.1)	1,043 (41.8)	462 (36.8)	0.006
By car	2,053 (54.7)	1,335 (53.4)	718 (57.1)	
By boat	21 (0.6)	16 (0.6)	5 (0.4)	
Mixed transport	176 (4.7)	104 (4.2)	72 (5.7)	
Time of travel to BMU, hours				
<1	3,039 (80.9)	2,054 (82.2)	985 (78.4)	<0.001
1–<3	610 (16.2)	397 (15.9)	213 (16.9)	
≥3	106 (2.8)	47 (1.9)	59 (4.7)	
Registration category				
New	3,337 (88.9)	2,248 (90.1)	1,089 (86.6)	0.002
Retreatment	416 (11.1)	248 (9.9)	168 (13.4)	
Site of TB disease				
Pulmonary	2,445 (65.1)	1,644 (65.8)	801 (63.7)	0.20
Extrapulmonary	1,310 (34.9)	854 (34.2)	456 (36.3)	
Type of TB by drug resistance				
DS-TB	3,135 (83.5)	2,097 (83.9)	1,038 (82.6)	0.065
DR-TB	17 (0.5)	7 (0.3)	10 (0.8)	
Clinical TB	603 (16.1)	394 (15.8)	209 (16.6)	
HIV status				
Positive	91 (2.4)	66 (2.6)	25 (2.0)	<0.001
Negative	2,750 (73.2)	1,979 (79.2)	771 (61.3)	
not tested	914 (24.3)	453 (18.1)	461 (36.7)	
TB confirmation method				
Bacteriologically confirmed	2,433 (64.8)	1,629 (65.2)	804 (64.0)	0.45
Clinical diagnosis	1,322 (35.2)	869 (34.8)	453 (36.0)	
TB treatment regimen					
First line	3,738 (99.5)	2,491 (99.7)	1,247 (99.2)	0.026
Second line	17 (0.5)	7 (0.3)	10 (0.8)	
Year treatment started					
2019	1,253 (33.4)	881 (35.3)	372 (29.6)	<0.001
2020	1,117 (29.7)	794 (31.8)	323 (25.7)	
2021	1,385 (36.9)	823 (32.9)	562 (44.7)	

PNG = Papua New Guinea; BMU = basic management unit; IQR = interquartile range; DS-TB= drug-susceptible TB; DR-TB = drug-resistant TB.

After adjusting for confounders through multivariable logistic regression, people who did not have an HIV test result were significantly more likely to have an unfavourable TB treatment outcome compared to HIV-negative patients (aOR 2.82, 95% CI 2.39–3.33) (Table 3). There were relatively few DR-TB cases (*n* = 23), but their odds of an unfavourable treatment outcome were significantly higher than DS-TB cases (aOR 3.26, 95% CI 1.18–9.00). Outcomes varied substantially by BMU site by year, so interaction terms improved model fit. Compared to treatments commenced in Modilon BMU in 2019, the odds of an unfavourable outcome were lower every year in Gaubin, not significantly different in any year in Malala or in Modilon in 2020, but outcomes were markedly worse in 2021 in Modilon (aOR 2.06, 95% CI 1.53–2.77). Combined time and mode of travel revealed specific associations with outcomes that were not evident without considering their interactions. Compared to travelling less than 1 hour by foot, people who had to travel for 1–<3 hours by foot had higher odds of an unfavourable treatment outcome (aOR 3.53, 95% CI 1.04–12.06), and travel ≥3 hours by car was also associated to a lesser extent with unfavourable outcomes (aOR 1.82, 95% CI 1.15–2.89).

**TABLE 3.** Factors associated with known unfavourable treatment outcomes in those who initiated treatment in Madang Province, 2019–2021.

Variables	OR (95% CI)	aOR 95% CI	*P*-value
Sex			
Male	1.00 (ref)	1.00 (ref)	
Female	0.88 (0.77–1.00)	0.88 (0.76–1.01)	0.0786
Age categories, years			
0–4	1.13 (0.88–1.46)	0.90 (0.69–1.18)	0.4472
5–14	0.99 (0.78–1.25)	0.89 (0.69–1.13)	0.3398
15–34	1.00 (ref)	1.00 (ref)	
35–64	0.85 (0.73–1.00)	0.85 (0.72–1.00)	0.0538
≥65	0.99 (0.66–1.48)	1.26 (0.81–1.96)	0.2955
Type of TB by drug resistance			
DS-TB	1.00 (ref)	1.00 (ref)	
DR-TB	2.89 (1.10–7.60)	3.26 (1.18–9.00)	0.0226
Clinical TB	1.07 (0.89–1.29)	0.94 (0.68–1.28)	0.6867
HIV status			
Positive	0.97 (0.61–1.55)	1.08 (0.67–1.74)	0.7588
Negative	1.00 (ref)	1.00 (ref)	
not tested	2.61 (2.24–3.05)	2.82 (2.39–3.33)	0.0000
Year treatment started and enrolling BMU*			
2019 Modilon	1.00 (ref)	1.00 (ref)	
2019 Gaubin	0.45 (0.34–0.61)	0.47 (0.32–0.69)	0.0001
2019 Malala	0.13 (0.02–0.97)	1.00	
2020 Modilon	0.91 (0.74–1.12)	1.07 (0.79–1.46)	0.6662
2020 Gaubin	0.55 (0.41–0.72)	0.57 (0.39–0.82)	0.0026
2020 Malala	0.82 (0.21–3.18)	0.86 (0.21–3.56)	0.8378
2021 Modilon	1.79 (1.49–2.16)	2.06 (1.53–2.77)	0.0000
2021 Gaubin	0.51 (0.39–0.68)	0.67 (0.46–0.97)	0.0333
2021 Malala	1.52 (0.41–5.71)	1.26 (0.31–5.11)	0.7481
Time and mode of travel to BMU*			
<1 h by foot	1.00 (ref)	1.00 (ref)	
<1 h by car	1.17 (1.00–1.37)	1.02 (0.87–1.21)	0.7752
<1 h by boat	2.28 (0.32–16.23)	1.83 (0.24–13.90)	0.5574
<1-h mixed transport	1.42 (0.92–2.20)	1.13 (0.71–1.78)	0.6049
1–<3 h by foot	3.19 (1.01–10.11)	3.53 (1.04–12.06)	0.0439
1–<3 h by car	1.18 (0.95–1.46)	1.18 (0.94–1.48)	0.1630
1–<3 h by boat	0.41 (0.09–1.88)	0.39 (0.08–1.97)	0.2562
1–<3 h mixed transport	1.54 (0.95–2.49)	1.50 (0.90–2.51)	0.1232
≥3 h by foot	1.00	1.00	
≥3 h by car	3.00 (1.94–4.64)	1.82 (1.15–2.89)	0.0112
≥3 h by boat	0.76 (0.08–7.32)	1.00 (0.10–9.73)	0.9993
≥3 h mixed transport	3.65 (1.19–11.21)	2.27 (0.71–7.27)	0.1684

* Interaction terms.

BMU = basic management unit, OR = odds ratio; CI = confidence interval; aOR = adjusted OR; DS-TB= drug-susceptible TB; DR-TB = drug-resistant TB.

Our findings were similar in a sensitivity analysis that included outcomes not evaluated as unfavourable, except for the associations with BMU by year treatment commenced. This was primarily due to relatively few outcomes not being evaluated in Modilon for treatments from 2021, when LTFU formed a substantially greater proportion of outcomes, and a relatively high number of unevaluated outcomes in Gaubin for treatments commenced in 2020. Malala BMU had a very high proportion of all outcomes not evaluated every year, indicating a consistent problem with completing outcome status.

## DISCUSSION

In this study, a high proportion of unfavourable treatment outcomes were observed from the three BMUs providing Xpert MTB/RIF testing in Madang Province over a 3-year period. Key factors that we found to be associated with unfavourable treatment outcomes were HIV status not tested, type of TB being DR-TB, and relatively long travel time to the BMU for the most common modes of travel (1–3 hours by foot or ≥3 hours by car). The study revealed differences by treatment facility that were not consistent over time.

The majority of TB patients in this study had been tested for HIV, which is an improvement compared to HIV testing coverage of only 36.6% reported for TB case notifications in Madang Province in 2016.^3^ In Gaubin, this has improved from 49% in 2014–2018 to 77%.^9^ However, coverage of HIV testing needs to increase further in Madang Province, as it does nationally. An unknown HIV status shows an important gap in adequate TB management, with a range of contributing factors potentially including healthcare worker knowledge, willingness or ability to offer and provide counselling and testing, and willingness of the patient to be tested due to stigma or lack of awareness. HIV status untested being associated with an unfavourable outcome may be a proxy for insufficient patient engagement by the health facility to support treatment completion and manage relevant comorbidities. People with DR-TB had a higher likelihood of unfavourable outcomes, which may indicate there had been insufficient clinical management and support to complete the historically longer and more challenging treatment regimens. The recently recommended shorter and more tolerable all-oral regimens should help,^15^ but patient education and support are still essential to achieve successful treatment outcomes.^16^ The population included in our study mainly resided within 1 hour of travel to the BMU facilities, but the additional time and distance for those who lived further away was a barrier to achieving a successful treatment outcome. Services may need to consider specific additional support for those whose access to the facility is particularly challenging. The differences in the outcomes by the facility and by year may relate to the different geographical settings, communities served, and facility capacities.

A key strength of the study was that all people registered for treatment over 3 years were included in the three BMUs in Madang Province. The BMUs serve urban, rural, and island populations and included approximately 90% of TB treatments provided in the province during this period, providing a good representation of the province. Although a relative proportion of cases remained undiagnosed, the proportion of cases detected via bacteriological confirmation highlights the recent introduction and nationwide expansion of Xpert testing to support microscopy testing for TB, which may have improved the case detection rates of TB and MDR/RR-TB.^17,18^ The data were limited by being extracted from routine treatment registers but were checked for validity and completeness to provide an accurate reflection of the register data.

An unacceptably high proportion of treatment outcomes were not evaluated in the treatment registers. The reasons for this are likely diverse but indicate that greater effort is needed to follow up patient treatment completion and record outcomes. Among those not evaluated, there may have been unrecorded LTFU or transfers between facilities that are unofficial or poorly recorded in stand-alone paper registers, as well as undetected deaths or failed treatments. The numbers and proportion of outcomes not evaluated due to patients transferring between facilities should be reduced by following the updated WHO guidance on TB surveillance that recommends that responsibility for reporting the final treatment outcome switches to the destination facility.^19^ Recent studies on LTFU in PNG presented similar findings in LMIC.^6,8,20^ One reason for the high proportions of not evaluated and LTFU outcomes could be that the Madang population is highly mobile, with high socio-economic activities and road linkage to other provinces. Treatment plans should pre-empt the relatively high likelihood of LTFU and provide strong patient education about the importance of treatment completion and supports that predict the patient's need for mobility during treatment. Studies have shown that improving patient support during treatment improves treatment success and reduces drug resistance.^21^ Patient treatment support remains a challenge in PNG; however, education and counselling involving counsellors and peer counsellors contributed to high retention in care for DS and DR-TB in the very high-incidence setting of Daru, PNG.^22,23^ While family members are not the preferred option for providing treatment support except for children,^24^ they can have a role in supporting the treatment journey to completion. Close contacts of pulmonary TB patients are at heightened risk of TB infection and disease.^25^ The provision of case finding and TB preventive treatment at the household level with family-integrated treatment support for disease and infection can bring multiple benefits, including improved treatment outcomes for the index case as well as reduced costs.

The unsatisfactory treatment outcomes reflect challenges for the NTP. Poor treatment success rates are observed throughout PNG and remain behind WHO estimates.^1^ Apart from geographical heterogeneity, the limited number of healthcare providers for TB may be a reason. Furthermore, the robustness of TB data to inform TB management is challenged by a lack of routine monitoring and evaluation activities in the province.

There are several potential solutions to address high rates of unsatisfactory treatment outcomes. The decentralisation of healthcare services through community engagement programmes, mobile health initiatives, capacity building programmes and stakeholder involvement are potentially important strategies to improve TB activities and reduce LTFU.^26,27^ Funding agencies and the NTP can play a role in strengthening social support systems. Providing financial incentives has been shown to reduce LTFU.^28^ Inclusive community practices and public awareness campaigns have also been shown to minimise stigma and improve treatment outcomes.^29^ Education programmes focused on TB prevention and treatment adherence using culturally relevant materials and community engagement strategies involving families, church groups and traditional healers as partners are all person-centred approaches in TB control programmes.^24,30^ Improving data management practices and establishing robust monitoring tools also have a role in improving treatment success and enhancing the quality of care for TB patients.^31^ Furthermore, implementing HIV testing within TB services in Madang Province could improve the uptake of HIV testing among TB patients, as was shown in population-based HIV impact assessment surveys in LMICs.^32^ This area needs to be adequately resourced to improve treatment outcomes and reduce the transmission of both diseases.^33^

Although not a focus of this study, the COVID-19 pandemic may have impacted case notifications and outcomes of people still on treatment when health services were acutely affected by COVID-19-focused activities in 2020 and 2021. The WHO 2022 Global TB Report noted that the impact of COVID-19 may have stalled TB's progress in meeting national and global targets.^1^

In conclusion, there are substantial challenges to improving TB services for the Madang Province population. This analysis has identified factors associated with known unfavourable treatment outcomes that can be potentially addressed in the delivery of person-centred care and treatment support. Potential solutions must be implemented and evaluated.
